# Targeted Temperature Management for Cardiac Arrest Due to Non-shockable Rhythm: A Systematic Review and Meta-Analysis of Randomized Controlled Trials

**DOI:** 10.3389/fmed.2022.910560

**Published:** 2022-06-03

**Authors:** Yi-Bing Zhu, Yan Yao, Yu Ren, Jing-Zhi Feng, Hui-Bin Huang

**Affiliations:** ^1^Department of Emergency, Guang'anmen Hospital, China Academy of Chinese Medical Sciences, Beijing, China; ^2^Department of Critical Care Medicine, School of Clinical Medicine, Beijing Tsinghua Changgung Hospital, Tsinghua University, Beijing, China

**Keywords:** non-shockable rhythm, cardiac arrest, targeted temperature management, neurological outcome, meta-analysis

## Abstract

**Background:**

Targeted temperature management (TTM) is recommended in adult patients following cardiac arrest (CA) with any rhythm. However, as to non-shockable (NSR) CA, high-quality evidence of TTM supporting its practices remains uncertain. Thus, we aimed to conduct a systematic review and meta-analysis with randomized controlled trials (RCTs) to explore the efficacy and safety of TTM in this population.

**Methods:**

We searched PubMed, Embase, and Cochrane library databases for potential trials from inception through Aug 25, 2021. RCTs evaluating TTM for CA adults due to NSR were included, regardless of the timing of cooling initiation. Outcome measurements were mortality and good neurological function. We used the Cochrane bias tools to evaluate the quality of the included studies. Heterogeneity, subgroup analyses, and sensitivity analysis were investigated to test the robustness of the primary outcomes.

**Results:**

A total of 14 RCTs with 4,009 adults were eligible for the final analysis. All trials had a low to moderate risk of bias. Of the included trials, six compared NSR patients with or without TTM, while eight compared pre-hospital to in-hospital TTM. Pooled data showed that TTM was not associated with improved mortality (Risk ratio [RR] 1.00; 95% CI, 0.944–1.05; *P* = 0.89, *I*^2^ = 0%) and good neurological outcome (RR 1.18; 95% CI 0.90–1.55; *P* = 0.22, *I*^2^ = 8%). Similarly, use of pre-hospital TTM resulted in neither an improved mortality (RR 0.99, 95% CI 0.97–1.03; *I*^2^ = 0%, *P* = 0.32) nor favorable neurological outcome (RR 1.13, 95% CI 0.93–1.38; *I*^2^ = 0%, *P* = 0.22). These results were further confirmed in the sensitivity analyses and subgroup analyses.

**Conclusions:**

Our results showed that using the TTM strategy did not significantly affect the mortality and neurologic outcomes in CA survival presenting initial NSR.

## Introduction

Cardiac arrest (CA) is a common public health problem with an estimated annual incidence rate of 28–55 per 100,000 person-years (1). Despite the advances in cardiopulmonary resuscitation (CPR) technology, the overall mortality rate is still high, up to 90% (2). Target temperature management (TTM) has been considered as an effective therapy to improve the neurological prognosis of comatose CA survivors after the return of spontaneous circulation (ROSC) (3). The mechanism may be related to the decrease in core body temperature, which reduces inflammation and cell damage after ischemia-reperfusion injury, and promotes the brain neurons healing by reducing cerebral oxygen demand and intracranial pressure (4). Thus, the use of TTM in CA survivors had been recommended consistently by published CPR guidelines (3, 5). However, the guidelines were challenged by the most recent trial conducted by Dankiewicz et al. (6), which concluded that in patients with coma after out-of-hospital cardiac arrest, targeted hypothermia did not improve survival or neurologic good outcome rates.

CA is a highly heterogeneous entity. Many factors will affect the effect of sub-hypothermia. Among them are the two types of presenting ECG rhythm in CA patients: a shockable rhythm (SR, ventricular fibrillation, or ventricular tachycardia) or a non-shockable rhythm (NSR, asystole, or pulseless electrical activity) during CA have received the most attention (7). Currently, the guidelines recommend TTM for CA survivors with SR or NSR (3). However, compared with CA survivors with SR having conclusive evidence of TTM to support their use, studies focusing on TTM for NSR survivors have reported conflicting results (7–9). Most current clinical recommendations are based on the consensus of expert opinions and extrapolate the potential benefits of TTM in NSR patients from the evidence of SR survivors (3, 4).

Recently, several high-quality RCTs evaluating the effect of TTM in CA patients have been published (6, 8, 10–13), and most of them focus on the subgroup of NSR survivors. Therefore, with the aid of the increased power of meta-analytic techniques, we aimed to review the relevant and available RCTs to describe the effectiveness of TTM in CA survivors with initial NSR.

## Methods

We conducted the current systematic review following the Preferred Reporting Items for Systematic Review and Meta-Analysis (PRISMA) statement (Appendix 1), and the review protocol had been published in the journal of Medicine (14).

### Eligibility Criteria

Studies were considered eligible if they investigated the efficacy and safety of TTM strategy in CA survivors presenting an initial NSR were included, regardless of the methods (evaporative cooling, infusion of cold saline, and surface or systemic cooling), timing (in-hospital or pre-hospital cooling), duration of TTM, or targeted temperature (32–36°C). We excluded studies conducted in neonates, children, pregnant, and studies that did not report data on survival. In addition, articles in abstract form without predefined data available or reviews or case series were also excluded.

### Search Strategy

We conducted a comprehensive systematic electronic search through PubMed, Cochrane library, and Embase databases from inception to Aug 15, 2021 (the last search) for potential RCTs, without language restriction. Boolean terms (OR and AND), Medical Subject Headings (MeSH), Emtree, and keywords were used in the search strategy. The search terms included “targeted temperature management,” “Therapeutic hypothermia,” “advanced cardiac life-support,” “cardiac arrest,” “cardiopulmonary resuscitation,” and “heart arrest.” The details of the research strategy was summarized in Appendix 2. Reference lists of relative articles were also manually checked to ensure the inclusion of all possible publications on this topic.

### Data Extraction and Quality Assessment

Two reviewers (Y-BZ and YY) extracted data independently from included studies on the first author' last name, publication year, study design (blinding or open-label; single or multi-centers), country where the study was conducted, study period, sample size, therapeutic regimens, follow-up duration, patient characteristics as well as all predefined outcomes. We appraised the risk of bias of the included RCTs using the Cochrane Collaboration tool for assessing the risk of bias (15). Discrepancies were identified and resolved through discussion.

### Outcome Measures

The primary outcome measure was mortality (considering the longest follow-up reported by the authors). The secondary outcome was good neurological function defined as a Cerebral Performance Category (CPC) score of 1 or 2. If trials only reported good neurological recovery, we considered this outcome to be CPC 1 or CPC 2 (16).

### Statistical Analysis

We pooled categorical data with risk ratios (RR) and continuous data with the mean difference (MD) using the Mantel-Haenszel, Inverse Variance fixed-effect, or Der Simonian and Laird random-effects model if needed. Some studies reported median as the measure of treatment effect, with accompanying interquartile range (IQR). Before data analysis, we estimated mean from median and standard deviations (SD) from IQR using the methods described in previous studies (17). Heterogeneity was quantified using the *I*^2^ statistic and its *P*-value. Studies with an *I*^2^ > 50% indicate significant heterogeneity (18).

To obtain more robust results, we estimated the pooled effect with their 95% CI if at least three studies with sufficient data available in each predefined outcome. Sensitivity analyses were performed by excluding trials that potentially biased the results. We further conducted subgroup analyses to test the robustness of the primary outcomes basing on the important clinical features (i.e., follow-up [short-term or long-term mortality; short-term mortality was defined as 28 days, ICU or hospital mortality, or mortality within 90 days of randomization, while long-term mortality was defined as a mortality rate of more than 180 days], by-stander CPR% [<50% or ≥50%], sample size [≥200 or <200], design [single-center or multi-centers], and OHCA% [100% or <100%]). Publication bias was evaluated by visually inspecting funnel plots. A two-sided *P* < 0.05 was considered statistically significant. All statistical analyses were performed using Review Manager, Version 5.3.

## Results

### Trial Identification

**Figure 1:**
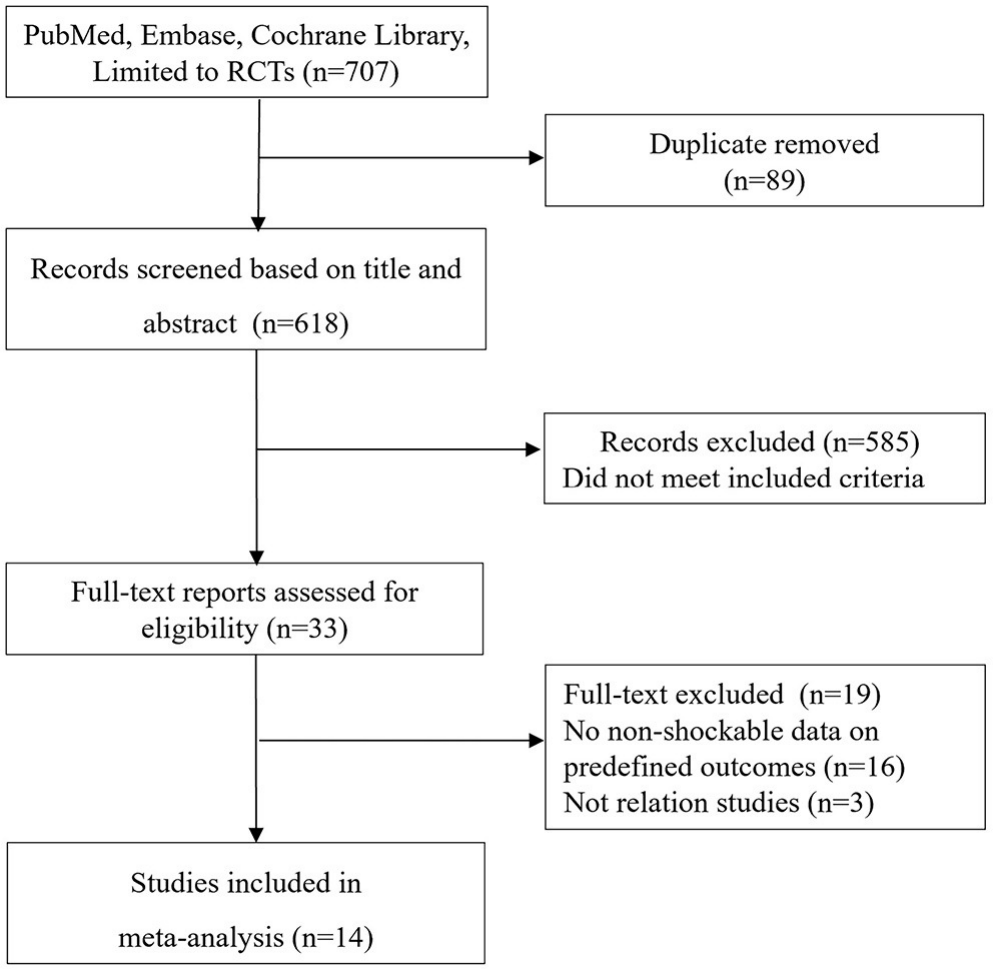
Selection process for studies included in the meta-analysis.

The de-duplicated results yielded 707 abstracts. The screen of abstracts and titles identified a total of 34 relevant studies for the following full-text review. Figure 1 shows a flowchart for the selection of studies. The excluded studies based on the full-text evaluation with exclusion reasons were summarized in Appendix 2. Finally, 14 RCTs (6, 10–13, 19–27) met the study inclusion criteria, of which six (6, 10, 11, 19–21) were comparing NSR patients with or without TTM while eight (12, 13, 22–27) were comparing NSR patients receiving pre-hospital or in-hospital TTM.

### Quality of the Studies

The quality of the included RCTs was low to moderate risk of bias (Appendix 3). However, funnel plots did not show skewed distributions, suggesting no publication bias was involved (Appendix 4).

### Study Characteristics

Tables 1, 2 shows the characteristics of the 14 included RCTs. Of these studies, 2 were single-center trials (19, 20), and 12 were multi-center trials (6, 10–13, 21–27). These RCTs were published between 2007 and 2021 from the France (*n* = 3), USA (*n* = 2), Australia (*n* = 2), Belgium (*n* = 2), Canada (*n* = 1), and multi-site (*n* = 4). A total of 4,009 NSR survivors were included in the final analysis (sample size ranging from 10 to 776 patients), with 2,022 patients in the TTM group and 1,987 patients in the non-TTM group. As to the initial rhythm, 11 RCTs included patients with SR or NSR (6, 12, 13, 19–21, 23–27), while the remaining three included only patients with NSR (10, 11, 22). Cooling methods varied among the RCTs, such as trans-nasal evaporative cooling, infusion of cold saline, and surface or systemic cooling.

**Table 1:** Characteristics of the studies included in current systemic review and meta-analysis.

**First author, year**	**Design**	**Research periods**	**Conducted country**	**Cooling method**	**TT (°C) TTM**	**TT(°C) Control**	**Sample size**	**OHCA (%)**	**Follow-up (days)**
Dankiewicz et al. (6)	P, OL, MC	2017–2020	Multi-sites	Mixed	33	<37.8°C	259/231	100	180
Lascarrou et al. (11)	P, OL, MC	2014–2018	France	Mixed	33	37°C	284/297	78	90
Frydland et al. (10)	P, OL, MC	2010–2013	Multi-sites	Mixed	33	36°C	96/82	100	180
Laurent et al. (21)	P, OL, MC	2000–2002	France	Systemic	32–33	No cooling	5/5	100	180
Hachimi-Idrissi et al. (20)	P, OL, SC	1999–2002	Belgium	External	33	37°C	17/16	100	180
Hachimi-Idrissi et al. (19)	P, OL, SC	1999–2000	Belgium	External	34	<38°C	16/14	100	14
Nordberg et al. (13)	P, OL, MC	2010–2018	Multi-sites	TNE	32–34	HC: 32–34°C	198/199	100	90
Scales et al. (27)	P, OL, MC	2012–2016	Canada	Cold saline/External	32–34	No cooling	155/169	100	Hospitalization
Bernard et al. (12)	P, OL, MC	2010–2014	Australia	Cold saline	33	No cooling	327/313	100	Hospitalization
Debaty et al. (24)	P, OL, MC	2009–2012	France	Cold saline/External	32–34	HC: 32–34°C	87/90	100	365
Bernard et al. (22)	P, OL, MC	2005–2007	Australia	Cold saline	33	HC: 32–34°C	82/81	100	Hospitalization
Castre'n et al. (23)	P, OL, MC	2008–2009	Multi-sites	TNE	34	HC: 34°C	66/69	100	Hospitalization
Kim et al. (25)	P, OL, MC	2004–2006	USA	Cold saline	<34	No cooling	34/40	100	Hospitalization
Kim et al. (26)	P, OL, MC	2007–2012	USA	Cold saline	<34	No cooling	396/380	100	Hospitalization

*CPR, cardiac pulmonary resuscitation; MC, multi-centers; NA, not available; OHCA, out-of-hospital cardiac arrest; OL, open-label; P, prospective; HC, hospital-cooling; SC, single-center; TT, targeted temperature; TNE, trans-nasal evaporative; TTM, target temperature management*.

**Table 2:** Characteristics of the patients included in current systemic review and meta-analysis.

**First author, year**	**TTM group/Control group**								
	**Sample size**	**Asystole%**	**PEA%**	**Bystander-CPR%**	**CA to ROSC time, minute**	**Age, year**	**Male,%**	**Mortality %**	**Good neurological outcome**
Dankiewicz et al. (9)	259/231	48/43	45/49	82/78	25/25	NA	NA	77/74	NA
Lascarrou et al. (11)	284/297	78/81	12/12	70/69	NA	67/67	65/63	81/83	13/14
Frydland et al. (10)	96/82	63	37	54	25/30	67	76	84/84	13/0
Laurent et al. (21)	5/5	100/87	0/13	NA	25/14	52/58	80/80	83/100	0/13
Hachimi-Idrissi et al. (20)	17/16	88/76	12/24	19/12	35/34	73/74	69/59	75/88	10/6
Hachimi-Idrissi et al. (19)	16/14	75/86	25/14	14/6	33/34	74/77	64/56	81/93	17/0
Nordberg et al. (13)	198/199	NA	NA	65/60	30/27	64/66	75/76	95/94	4/5
Scales et al. (27)	155/169	NA	NA	44/48	NA	68/69	70/61	92/92	14/13
Bernard et al. (12)	327/313	62/61	37/38	66/67	NA	65/64	75/74	98/99	NA
Debaty et al. (24)	87/90	91/90	9/10	50/52	27/30	66/69	72/71	98/99	NA
Bernard et al. (22)	82/81	50/37	50/63	44/38	29/29	64/61	69/59	87/89	12/9
Castre'n et al. (23)	66/69	71/67	29/33	36/46	32/30	66/64	72/78	94/96	25/14
Kim et al. (25)	34/40	NA	NA	67/74	NA	67/65	67/74	94/80	NA
Kim et al. (26)	396/380	53/53	44/48	54/52	NA	68/68	55/54	81/84	29/25

*CA, cardiac arrest; CPR, cardiac pulmonary resuscitation; NA, not available; OHCA, out-of-hospital cardiac arrest; PEA, pulseless electrical activity; ROSC, return of spontaneous circulation; TTM, target temperature management*.

### Outcomes

#### With or Without TTM

**Figure 2:**
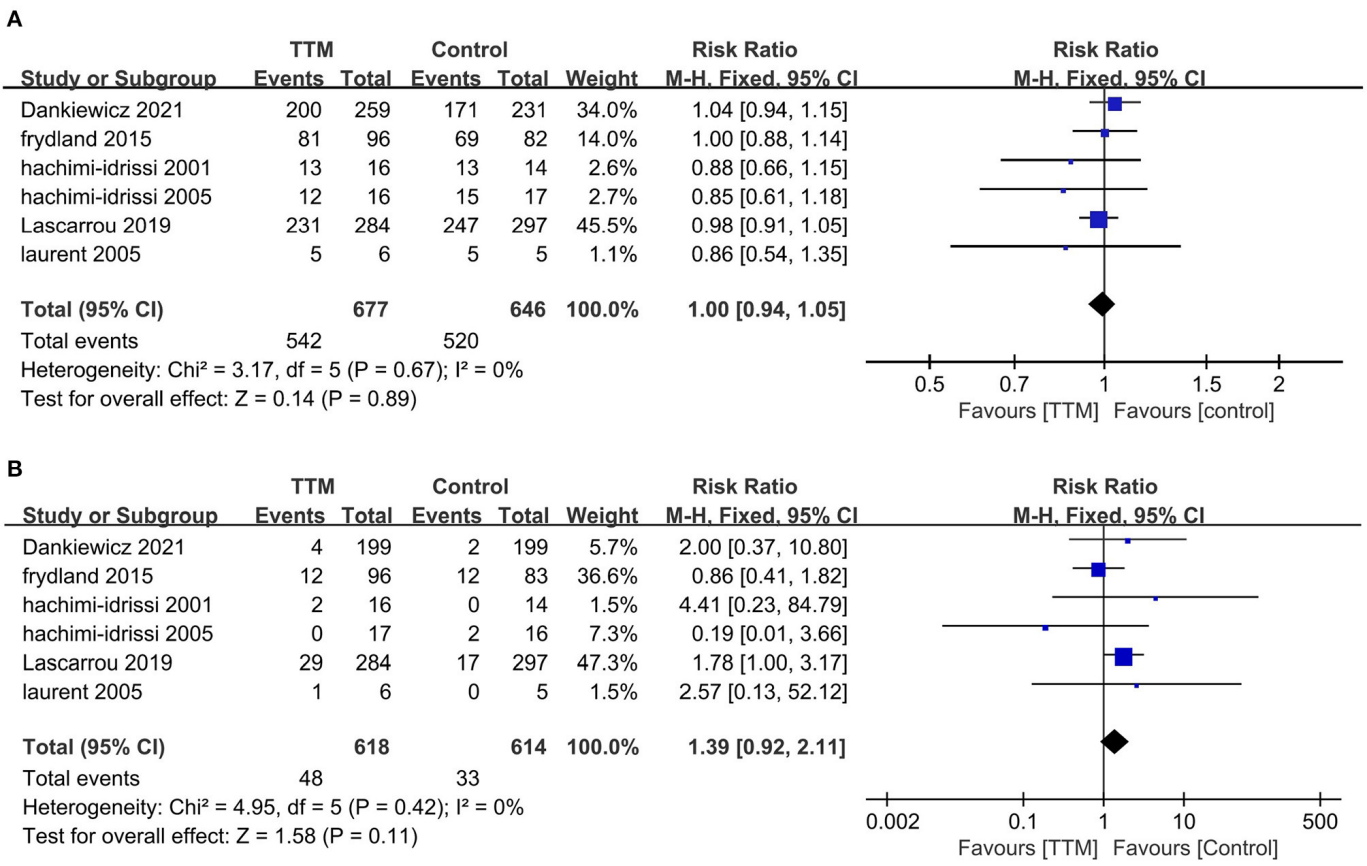
Forest plot of the non-shockable rhythm survivors with or without targeted temperature management. **(A)** Mortality; **(B)** Good neurological outcome.

Six studies compared NSR survivors with or without TTM, and all reported outcomes of mortality (6, 10, 11, 19–21). Of the 677 patients in the TTM group, 542 died, compared to 520 of 646 patients in the non-TTM group. The pooled analysis suggested TTM did not affect the mortality (RR = 1.00; 95% CI, 0.944–1.05; *P* = 0.89, *I*^2^ = 0%) (Figure 2A). Five studies focused on neurological outcomes as interests (10, 11, 19–21). Pooled analysis showed the good neurological outcome was comparable between the TTM group and non-TTM groups (*n* = 1,232; RR 1.39; 95% CI 0.92–2.11; *P* = 0.11, *I*^2^ = 0%) (Figure 2B).

In the sensitivity analysis, excluding any single test did not significantly change the overall combined OR of the survival (*P*-value ranged from 0.21 to 0.87) and neurological outcomes (*P*-value ranged from 0.19 to 0.98). Similarly, subgroup analyses based on study design, sample size, country, or initial rhythm showed no differences in the survival outcomes and good neurological outcomes (Table 2) between the TTM and non-TTM groups (Table 3).

**Table 3:** Subgroup analysis of the primary outcome based on TTM strategy.

		**Studies number**	**Patient, number**	**Event in the intervention group**	**Event in the control group**	**Risk ratio** **(95% CI)**	***I* ^2^**	***P***
With or without TTM		**6**	**1323**	**TTM group**	**Non-TTM group**	1.00 [0.94, 1.05]	0%	0.90
Follow-up	Short-term	3	219	99 of 118	87 of 101	0.97 [0.87, 1.09]	0%	0.66
	Long-term	3	1099	437 of 557	426 of 542	1.00 [0.94, 1.06]	0%	0.97
Sample size	>200	2	1071	431 of 543	418 of 528	1.01 [0.95, 1.07]	3%	0.85
	<200	4	247	105 of 132	95 of 115	0.86 [0.71, 1.05]	16%	0.16
Design	MC	4	652	517 of 645	492 of 615	1.00 [0.95, 1.06]	0%	0.90
	SC	2	63	25 of 32	28 of 31	0.86 [0.69, 1.07]	0%	0.18
OHCA%	<100	1	581	231 of 284	247 of 297	0.98 [0.91, 1.05]	–	–
	100	5	1249	512 of 639	487 of 610	1.01 [0.95, 1.06]	0%	0.85
Bystander CPR%	≥50	3	1249	512 of 639	487 of 610	1.01 [0.95, 1.06]	0%	0.85
	<50	3	74	30 of 38	33 of 36	0.86 [0.71, 1.05]	0%	0.14
Pre- or In-hospital TTM		**8**	**2686**	**Pre-hospital TTM**	**In-hospital TTM**	0.99 [0.97, 1.01]	0%	0.85
Follow-up	Short-term	8	2686	1220 of 1345	1231 of 1341	0.99 [0.97, 1.01]	0%	0.85
	Long-term	–	–	–	–	–	–	–
Sample size	>200	4	2137	970 of 1076	970 of 1061	0.99 [0.96, 1.01]	0%	0.31
	<200	4	549	250 of 269	261 of 280	1.00 [0.95, 1.04]	34%	0.88
Design	MC	8	2686	1220 of 1345	1231 of 1341	0.99 [0.97, 1.01]	0%	0.85
	SC	–	–	–	–	–	–	–
Bystander CPR%	≥50	5	2064	945 of 1042	936 of 1022	0.99 [0.97, 1.02]	21%	0.48
	<50	3	622	275 of 303	295 of 319	0.98 [0.94, 1.03]	0%	0.45
Bystander CPR%	≥50	8	2686	1220 of 1345	1231 of 1341	0.99 [0.97, 1.01]	0%	0.85
	<50	–	–	–	–	–	–	–

*CPR, cardiac-pulmonary resuscitation; OHCA, out-of-hospital cardiac arrest; TTM, targeted temperature management*.

#### Pre-hospital or In-hospital TTM

**Figure 3:**
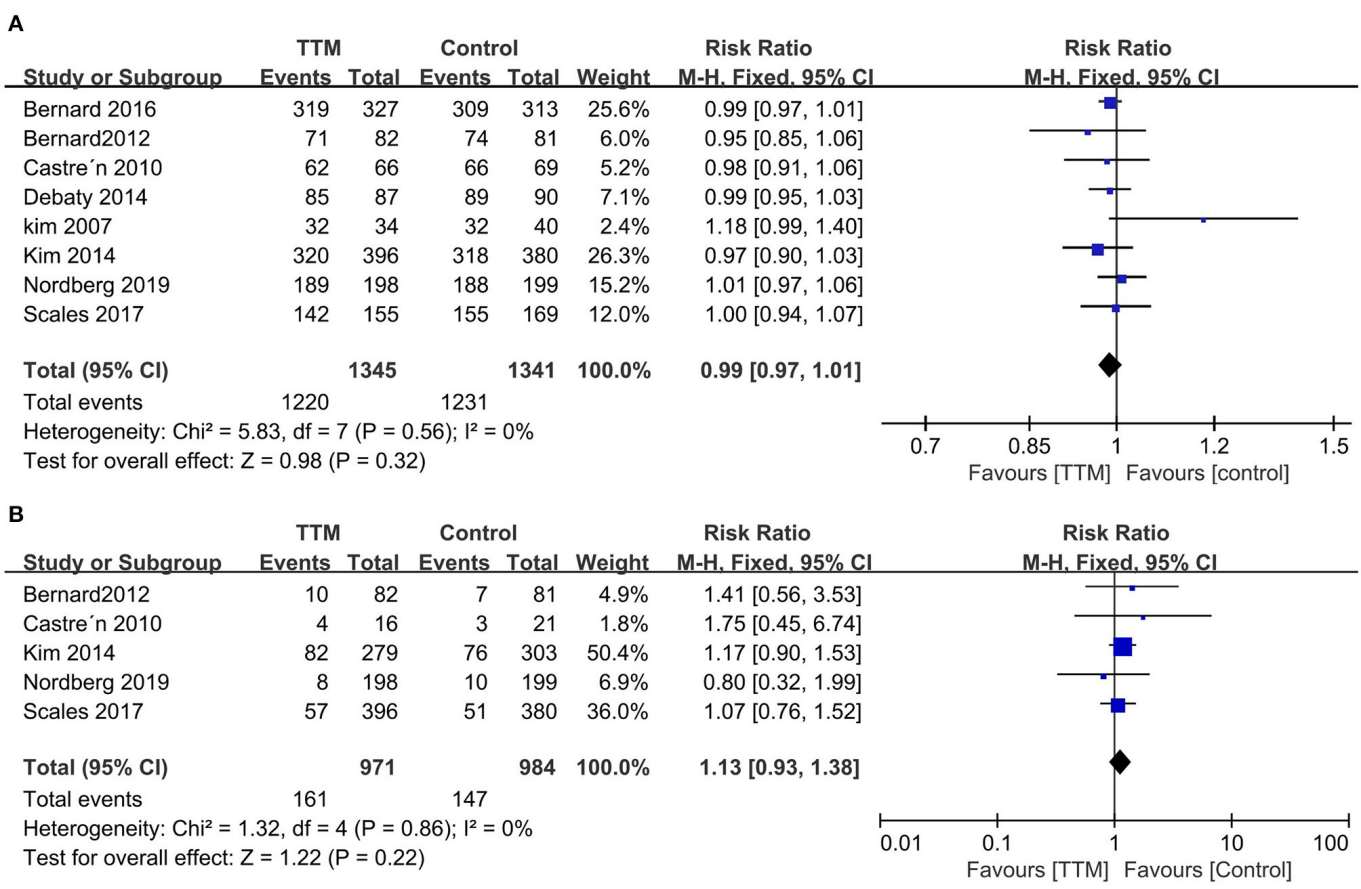
Forest plot of the targeted temperature management used in pre-hospital or in-hospital survivors with non-shockable rhythm. **(A)** Mortality; **(B)** Good neurological outcome.

All eight trials reported outcome of mortality (1,345 in pre-hospital group and 1,341 in in-hospital group) (12, 13, 22–27). The pooled mortality rate was similar when we compared the pre-hospital TTM group with the in-hospital TTM group (8 trials, *N* = 2,682; RR 0.99, 95% CI 0.97–1.01, *I*^2^ = 0) (Figure 3A). Five RCTs focused on neurological outcomes as interests. When pooled, the result showed no difference in favorable neurological outcome (6 trials, *N* = 1,955; RR 1.13, 95% CI 0.93–1.18, *I*^2^ = 0) (Figure 3B).

## Discussion

In the current meta-analysis, we included 14 RCTs focusing on CA survivors with NSR to evaluate the prognosis of TTM for these patients. Most included studies were low to medium risk of bias in quality. Our results showed that TTM did not significantly improve survival or neurological outcomes in CA patients with NSR than those who did not. Meanwhile, early starting cooling strategies such as prehospital TTM showed no more benefits in such a patient population. These results were further supported by subgroup analysis and sensitivity analysis.

### Comparison With Previous Studies

Our results showed that many CA survivors with NSR received TTM despite no high-quality evidence to support its use. This may be due to the recommendations of the CPR guidelines (3, 5), which had consistently recommended TTM for CA survivors with both SR and NSR. However, the recommendation for the NSR patients is mainly based on an extrapolation of SR patients but no clear evidence. The latest 2021 guideline has been modified to suggest TTM for adult CA patients with initial NSR who remain unresponsive after ROSC (3). However, it is still a weak recommendation and is from very low-quality evidence.

So far, there have been 3 meta-analyses published on this topic with inconsistent results (7–9). However, the main problem of these articles was the inclusion of most observational studies but only a small number of RCTs, leading to a high risk of bias and random error in these studies. To address the limitations of the previous meta-analysis, we only included RCTs and added several recent published trials, with a total sample size of 4,009 patients in the current study (6, 10–13, 19–27). Thus, our sample size allowed for better statistical power and other sensitivities and subgroup analyses. The results of subgroup and sensitivity analyses for ICHA initial rhythm, study start date, study design, OHCA%, and recent and long-term prognosis corroborated the robustness of our findings. Thus, our results provide conclusive evidence regarding the impact of TTM on mortality and neurologic outcomes of CA survivors with initial NSR.

It is also worth noting that the International Liaison Committee on Resuscitation (ILCOR) recently published a systematic review that there was no improvement in survival or favorable neurologic outcome in TTM groups compared with normothermia groups. The prehospital cooling groups also showed no benefits in survival or favorable neurologic outcome in comparison with on prehospital cooling groups. These findings may warrant an update of international cardiac arrest guidelines (28).

### Explaining Our Findings

We found TTM did not benefit CA patients with initial NSR concerning the mortality and neurological prognosis. Some might contribute to this. On the one hand, NSR has a marked impact on the CA prognosis, not only the reduced chances of obtaining ROSC but also the chances of surviving hospital discharge. The CA patients' leading cause of death is the neurological injury from anoxic brain damage, independent of initial rhythm (29, 30). The potential mechanism of TTM included anti-oxidant, anti-apoptotic and anti-inflammatory effects and a decrease in the accumulation or release of excitotoxic amino acids (4, 28, 31). The positive results from animal studies and trials led to the inclusion of TTM in the guidelines (32, 33). However, NSR survivors have more poor prognostic factors than those of SR (26, 34). As shown in our results, NSR survivors were usually older, had more comorbidities, and suffered an increased risk of multiple organ dysfunction. Thus, those risks might partly weaken the advantages of TTM application.

On the other hand, the included studies spanned an extensive range of periods, during which CPR and CA guidelines have been updated several times (3, 5). CA prognosis might benefit from several improved techniques such as bystander intervention, advanced cardiac life support, emergency cardiac catheterization, and optimal support for brain functions (3). Therefore, these techniques may limit the theoretical benefits of TTM to reduce free radical-mediated reperfusion injury in hypoxic brain injury.

Additionally, the prehospital cooling strategy also showed no more prognosis benefits in NSR patients after CA than those receiving TTM after hospital arrival. Several reasons might help explain the negative findings. First, prehospital cooling patients have more re-arrest episodes and pulmonary edema due to the infusion of a large amount of cold intravenous saline immediately after ROSC (26). Meanwhile, the rapid infusion can also cause an increase in right atrium pressure, which may reduce coronary perfusion pressure and, therefore, myocardial perfusion (35). Second, the drop in temperature by rapid infusion of cold saline might be too slight. As shown in the study by Bernard et al., the authors found no differences in outcomes when cooling was initiated before hospital arrival between the groups (12). Third, prehospital cooling did not significantly reduce the time to targeted temperature. Scales et al. (27) reported that achieving a target temperature of <34°C within 6-h of hospital arrival was not significantly different between CA survivors with or without prehospital cooling patients. In addition, studies have shown that lower myocardial temperature increases the rate of successful defibrillation. However, this does not affect patients with NSR (36).

### Research Limitations

Our study has several limitations. First, all of the included RCTs are open-label designs. This might result in the selection bias of our research. Second, CA is a highly heterogeneous entity, and many factors may affect the efficacy of TTM, such as the cooling methods, sedative drugs, timing, and shivering monitoring methods. Third, although we had used subgroup analyses and sensitivity analysis to explore the possible confounding factors, our results may be affected by unmeasured factors. Fourth, prognostic assessment methods varied among the included studies. Some clinicians chose telephone interviews rather than face-to-face interviews. Fifth, the non-TTM CA survivors varied in the temperature management, such as target 36°C to <38°C or no cooling, which may affect the robustness of our conclusions. Finally, the included CA patients have different underlying diseases, demographic characteristics and use different disease severity scoring standards. However, due to the number of studies, we cannot further perform subgroup analysis to clarify this point.

## Conclusions

Among patients with NSR, the use of TTM showed no more benefits than usual care in survival and favorable neurological outcomes. However, our results were based on observational findings and warranted a randomized clinical trial to assess the efficacy of TTM for such a patient population.

## Data Availability Statement

The original contributions presented in the study are included in the article/Supplementary Material, further inquiries can be directed to the corresponding author/s.

## Author Contributions

Y-BZ contributed to conception and design and drafted the manuscript. YY and Y-BZ contributed to searching the scientific literature and data interpretation. J-ZF and YR helped to collect the data and performed statistical analyses. H-BH was responsible for the integrity of the work as a whole, from inception to publication of the article. All authors read and approved the manuscript.

## Funding

This research was supported by the Science and Technological Innovation Project of China Academy of Chinese Medical Sciences (CACMS) Innovation Fund (CI2021A02904).

## Conflict of Interest

The authors declare that the research was conducted in the absence of any commercial or financial relationships that could be construed as a potential conflict of interest.

## Publisher's Note

All claims expressed in this article are solely those of the authors and do not necessarily represent those of their affiliated organizations, or those of the publisher, the editors and the reviewers. Any product that may be evaluated in this article, or claim that may be made by its manufacturer, is not guaranteed or endorsed by the publisher.

## Abbreviations

CA: cardiac arrest
CI: confidence interval
ICU: intensive care unit
IHCA: in-hospital cardiac arrest
ILCOR: International Liaison Committee on Resuscitation
IQR: interquartile range
LOS: length of stay
MD: mean difference
NSR: non-shockable rhythm
OHCA: out-of-hospital cardiac arrest
ROSC: return of spontaneous circulation
RR: risk ratio
RCTs: randomized controlled trials
SD: standard deviations
SR: shockable rhythm
TTM: targeted temperature management.
