# Supplementary material for: Targeted Temperature Management for Cardiac Arrest Due to Non-shockable Rhythm: A Systematic Review and Meta-Analysis of Randomized Controlled Trials
Source: Front Med (Lausanne). 2022 Jun 3;9:910560. doi: 10.3389/fmed.2022.910560 (PMC9203727; doi:10.3389/fmed.2022.910560)
Supplement: Supplementary file 1 [file Data_Sheet_1.DOCX]

**Targeted temperature management for cardiac arrest due to non-shockable rhythm: a systematic review and meta-analysis of randomized controlled trials**

**Additional files**

Additional file 1 PRISMA checklist……………………………………………………………………………………………………………………………………………………………………………………………….……………………………………..2

Additional file 2 Search Strategy………………………………………………………………………………………………………………………………………………………………………………………………………………….……………………..5

Additional file 3 Fig: Definition of inclusion and exclusion criteria for patient population and regimens of conservative and conventional oxygen…………………………………………………...……..8

Additional file 4 Fig: Cochrane risk of bias………………………………………………………………………………………………………………………………………………………………………………………………………………....……..10

Additional file 6 Table: Adverse events..………………………………..…………………………………………………….………………………………………………………………………………..………………………………….…………....…11

**Additional file 1**

**PRISMA 2009 checklist**

| **Section/topic** | **#** | **Checklist item** | **Reported on page #** |
| --- | --- | --- | --- |
| **TITLE** | | |  |
| Title | 1 | Identify the report as a systematic review, meta-analysis, or both. | 1 |
| **ABSTRACT** | | |  |
| Structured summary | 2 | Provide a structured summary including, as applicable: background; objectives; data sources; study eligibility criteria, participants, and interventions; study appraisal and synthesis methods; results; limitations; conclusions and implications of key findings; systematic review registration number. | 2 |
| **INTRODUCTION** | | |  |
| Rationale | 3 | Describe the rationale for the review in the context of what is already known. | 4 |
| Objectives | 4 | Provide an explicit statement of questions being addressed with reference to participants, interventions, comparisons, outcomes, and study design (PICOS). | 4-5 |
| **METHODS** | | |  |
| Protocol and registration | 5 | Indicate if a review protocol exists, if and where it can be accessed (e.g., Web address), and, if available, provide registration information including registration number. | 6 |
| Eligibility criteria | 6 | Specify study characteristics (e.g., PICOS, length of follow-up) and report characteristics (e.g., years considered, language, publication status) used as criteria for eligibility, giving rationale. | 6 |
| Information sources | 7 | Describe all information sources (e.g., databases with dates of coverage, contact with study authors to identify additional studies) in the search and date last searched. | 6 |
| Search | 8 | Present full electronic search strategy for at least one database, including any limits used, such that it could be repeated. | 6 and Appendix 2 |
| Study selection | 9 | State the process for selecting studies (i.e., screening, eligibility, included in systematic review, and, if applicable, included in the meta-analysis). | 6 |
| Data collection process | 10 | Describe method of data extraction from reports (e.g., piloted forms, independently, in duplicate) and any processes for obtaining and confirming data from investigators. | 6-7 |
| Data items | 11 | List and define all variables for which data were sought (e.g., PICOS, funding sources) and any assumptions and simplifications made. | 7 |
| Risk of bias in individual studies | 12 | Describe methods used for assessing risk of bias of individual studies (including specification of whether this was done at the study or outcome level), and how this information is to be used in any data synthesis. | 7 |
| Summary measures | 13 | State the principal summary measures (e.g., risk ratio, difference in means). | 7 |
| Synthesis of results | 14 | Describe the methods of handling data and combining results of studies, if done, including measures of consistency (e.g., I^2^) for each meta-analysis. | 7-8 |

| Risk of bias across studies | 15 | Specify any assessment of risk of bias that may affect the cumulative evidence (e.g., publication bias, selective reporting within studies). | 8 |
| --- | --- | --- | --- |
| Additional analyses | 16 | Describe methods of additional analyses (e.g., sensitivity or subgroup analyses, meta-regression), if done, indicating which were pre-specified. | 8 |
| **RESULTS** | | |  |
| Study selection | 17 | Give numbers of studies screened, assessed for eligibility, and included in the review, with reasons for exclusions at each stage, ideally with a flow diagram. | 9 |
| Study characteristics | 18 | For each study, present characteristics for which data were extracted (e.g., study size, PICOS, follow-up period) and provide the citations. | 9, Table 1,  Appendix 3 |
| Risk of bias within studies | 19 | Present data on risk of bias of each study and, if available, any outcome level assessment (see item 12). | 9  Appendix 4 |
| Results of individual studies | 20 | For all outcomes considered (benefits or harms), present, for each study: (a) simple summary data for each intervention group (b) effect estimates and confidence intervals, ideally with a forest plot. | 9 |
| Synthesis of results | 21 | Present results of each meta-analysis done, including confidence intervals and measures of consistency. | 9-11 |
| Risk of bias across studies | 22 | Present results of any assessment of risk of bias across studies (see Item 15). | Appendix 4 |
| Additional analysis | 23 | Give results of additional analyses, if done (e.g., sensitivity or subgroup analyses, meta-regression [see Item 16]). | 10-11  Table 2-3 |
| **DISCUSSION** | | |  |
| Summary of evidence | 24 | Summarize the main findings including the strength of evidence for each main outcome; consider their relevance to key groups (e.g., healthcare providers, users, and policy makers). | 12-15 |
| Limitations | 25 | Discuss limitations at study and outcome level (e.g., risk of bias), and at review-level (e.g., incomplete retrieval of identified research, reporting bias). | 15 |
| Conclusions | 26 | Provide a general interpretation of the results in the context of other evidence, and implications for future research. | 17 |
| **FUNDING** | | |  |
| Funding | 27 | Describe sources of funding for the systematic review and other support (e.g., supply of data); role of funders for the systematic review. | 18 |

**Additional file 2**

**Search Strategy：**

**Database: PubMed, Embase, Cochrane library；**

**Search completed on 15th Jul 2021.**

=====================================================================================================================

**PubMed** 498

Search: (((((("death, sudden, cardiac"[MeSH Terms]) OR ("Out-of-Hospital Cardiac Arrest"[MeSH Terms])) OR ("Heart Arrest"[MeSH Terms])) OR ("cardiac arrest"[Title/Abstract])) AND ((("target temperature management"[Title/Abstract]) OR ("therapeutic hypothermia"[Title/Abstract])) OR ("hypothermia, induced"[MeSH Terms]))) AND ((randomized controlled trial [pt] OR controlled clinical trial [pt] OR randomized [tiab] OR placebo [tiab] OR clinical trials as topic [mesh: noexp] OR randomly [tiab] OR trial [ti]) NOT (animals [mh] NOT humans [mh]))) AND ((("non-shockable rhythm") OR (asystole)) OR ("pulseless electrical activity")) Sort by: Most Recent **-------------------------------------------------------------------------------------------------------------------------------------------------------------------------------------------------------**

**Embase** 75

No. Query Results

#14. #4 AND #12 AND #13 AND #8

#13. 'clinical trial'/exp OR ' randomization '/exp OR 'randomized controlled trial'/exp OR 'single blind procedure'/exp OR 'double blind procedure'/exp OR 'randomized controlled trial'/exp OR 'crossover procedure'/exp OR 'placebo'/exp OR 'prospective studies'/exp OR 'randomi?ed controlled' NEXT/1 trial* OR RCT OR 'randomly allocated' OR 'allocated randomly' OR ' random allocation' OR allocated NEAR/2 random OR single NEXT/1 blind* OR double NEXT/1 blind* OR (treble OR triple) NEAR/1 blind* OR placebo*

#12. #9 OR #10 OR #11

#11. 'pulseless electrical activity':ab,ti

#10. 'asystole':ab,ti

#9. 'non-shockable rhythm':ab,ti

#8. #5 OR #6 OR #7

#7. 'heart arrest':ab,ti

#6. 'cardiac arrest':ab,ti

#5. 'heart arrest'/exp

#4. #1 OR #2 OR #3

#3. 'induced hypothermia'/exp

#2. 'therapeutic hypothermia':ab,ti

#1. 'target temperature management':ab,ti

---------------------------------------------------------------------------------------------------------------------------------------------------------------------------------------------------------

**Cochrane library** 134

ID Search

#1 target temperature management

#2 MeSH descriptor: [Hypothermia, Induced] explode all trees

#3 therapeutic hypothermia

#4 #1 OR #2 OR #3

#5 Cardiac Arrest

#6 MeSH descriptor: [Heart Arrest] explode all trees

#7 heart arrest

#8 death, sudden, cardiac

#9 MeSH descriptor: [Death, Sudden, Cardiac] explode all trees

#10 #5 OR #6 OR #7 OR #8 OR #9

#11 pulseless electrical activity

#12 asystole

#13 non-shockable rhythm

#14 #11 OR #12 OR # 13

#15 #4 AND #10AND #14

**Additional file 3: Cochrane risk of bias**

**Risk of bias summary: review authors' judgements about each risk of bias item for each included study.**

| **Study** | **Sequence generation** | **Allocation concealment** | **Blinding*** | **Incomplete outcome data** | **Selective outcome reporting** | **Other sources of bias** | **Risk assessment** |
| --- | --- | --- | --- | --- | --- | --- | --- |
| Dankiewicz 2021 | Low | Low | Low | Low | Low | Low | Low |
| Lascarrou 2019 | Low | Low | Low | Low | Low | Low | Low |
| Frydland 2015 | Low | Low | Low | Low | Low | Unclear | Low |
| Laurent 2005 | Low | Low | Low | Low | Low | Unclear | Low |
| Hachimi-Idrissi 2004 | Unclear | Unclear | Unclear | low | low | Unclear | Moderate |
| Hachimi-Idrissi 2001 | Unclear | Unclear | Unclear | low | low | Unclear | Moderate |
| Nordberg 2019 | Low | Low | Low | Low | Low | Low | Low |
| Scales 2017 | Low | Low | Low | Low | Low | Unclear | Low |
| Bernard 2016 | Low | Low | Low | Low | Low | Unclear | Low |
| Debaty 2014 | Low | Low | Unclear | Low | Low | Unclear | Moderate |
| Bernard 2012 | Low | Low | Unclear | Low | Low | High | High |
| Castre´n 2010 | Low | Low | Unclear | Low | Low | Unclear | Moderate |
| Kim 2007 | Unclear | Unclear | Unclear | low | low | Unclear | Moderate |
| Kim 2014 | Low | Unclear | Unclear | Low | Low | Unclear | Moderate |

*As blinding of caregivers, patients, and family members was impossible in these trials, we considered blinding only at the data collection level.

**Additional file 4: Funnel plot of comparison: mortality at the longest follow-up available**


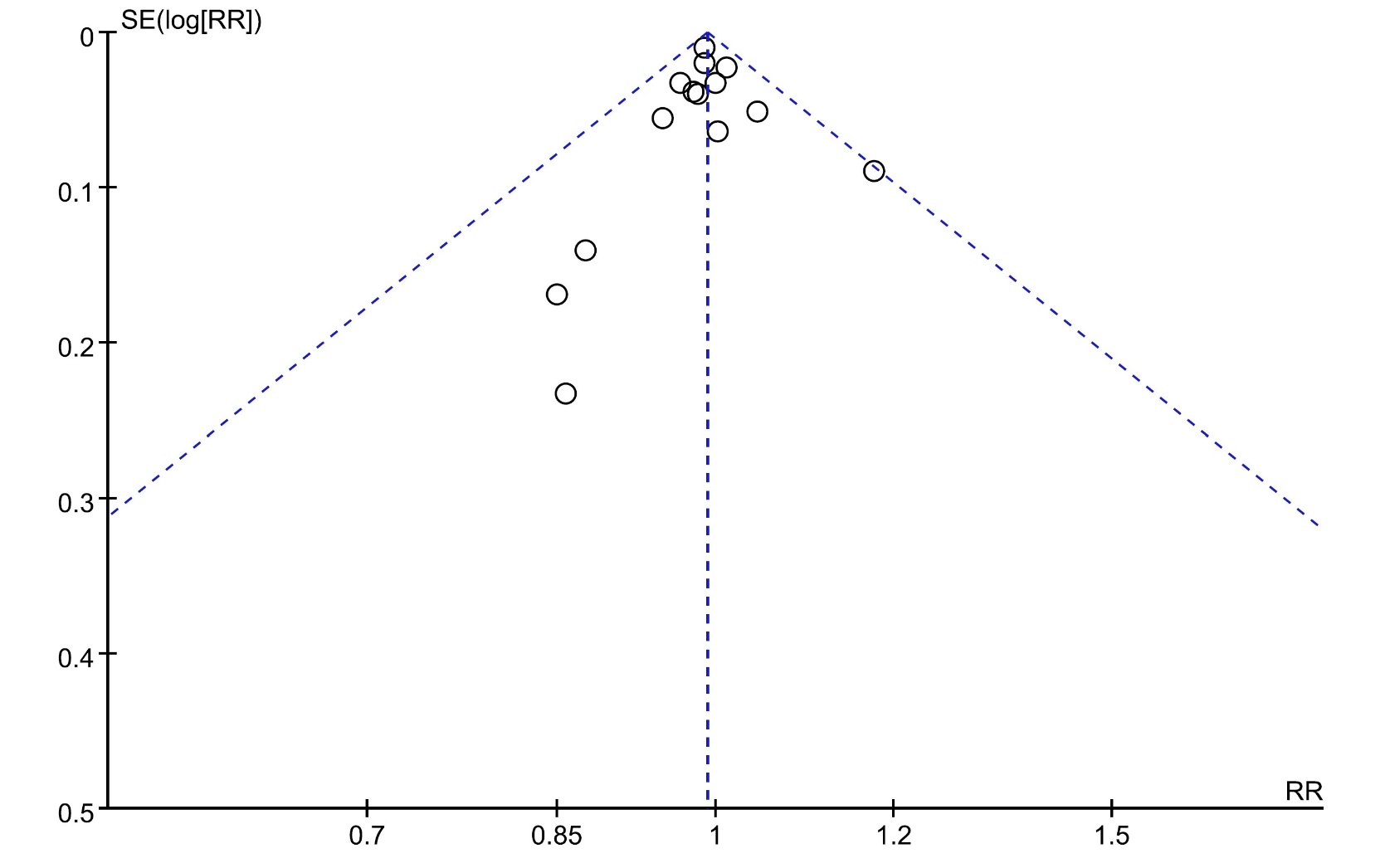


**Figure**: Funnel plot of comparison: weaning rate based on mortality.
